# Combined Transcriptomic and Proteomic Profiling to Unravel Osimertinib, CARP-1 Functional Mimetic (CFM 4.17) Formulation and Telmisartan Combo Treatment in NSCLC Tumor Xenografts

**DOI:** 10.3390/pharmaceutics14061156

**Published:** 2022-05-28

**Authors:** Ramesh Nimma, Anil Kumar Kalvala, Nilkumar Patel, Sunil Kumar Surapaneni, Li Sun, Rakesh Singh, Ebony Nottingham, Arvind Bagde, Nagavendra Kommineni, Peggy Arthur, Aakash Nathani, David G. Meckes, Mandip Singh

**Affiliations:** 1 College of Pharmacy and Pharmaceutical Sciences, Florida A&M University, Tallahassee, FL 32307, USA; ramesh.nimma@famu.edu (R.N.); anil.kalvala@famu.edu (A.K.K.); nealpatel442@gmail.com (N.P.); sunil30.niper@gmail.com (S.K.S.); ebony1.nottingham@famu.edu (E.N.); bagde.arvind02@gmail.com (A.B.); nagavendra.kommineni@famu.edu (N.K.); peggyart33@gmail.com (P.A.); aakash1.nathani@famu.edu (A.N.); 2 Department of Biomedical Sciences, College of Medicine, Florida State University, 1115 West Call Street, Tallahassee, FL 32306, USA; li.sun@med.fsu.edu (L.S.); david.meckes@med.fsu.edu (D.G.M.J.); 3 Department of Translational Science Laboratory, College of Medicine, Florida State University, 1115 West Call St., Tallahassee, FL 32306, USA; rsingh3@fsu.edu

**Keywords:** epidermal growth factor receptor, non-small cell lung cancer, Lamin B2, AMPK, Osimertinib, RNA seq, proteomics, RT-PCR

## Abstract

The epidermal growth factor receptor (EGFR) is highly expressed in many non-small cell lung cancers (NSCLC), necessitating the use of EGFR-tyrosine kinase inhibitors (TKIs) as first-line treatments. Osimertinib (OSM), a third-generation TKI, is routinely used in clinics, but T790M mutations in exon 20 of the EGFR receptor lead to resistance against OSM, necessitating the development of more effective therapeutics. Telmisartan (TLM), OSM, and cell cycle and apoptosis regulatory protein 1 (CARP-1) functional mimetic treatments (CFM4.17) were evaluated in this study against experimental H1975 tumor xenografts to ascertain their anti-cancer effects. Briefly, tumor growth was studied in H1975 xenografts in athymic nude mice, gene and protein expressions were analyzed using next-generation RNA sequencing, proteomics, RT-PCR, and Western blotting. TLM pre-treatment significantly reduced the tumor burden when combined with CFM-4.17 nanoformulation and OSM combination (TLM_CFM-F_OSM) than their respective single treatments or combination of OSM and TLM with CFM 4.17. Data from RNA sequencing and proteomics revealed that TLM_CFM-F_OSM decreased the expression of Lamin B2, STAT3, SOD, NFKB, MMP-1, TGF beta, Sox-2, and PD-L1 proteins while increasing the expression of AMPK proteins, which was also confirmed by RT-PCR, proteomics, and Western blotting. According to our findings, the TLM_CFM-F_OSM combination has a superior anti-cancer effect in the treatment of NSCLC by affecting multiple resistant markers that regulate mitochondrial homeostasis, inflammation, oxidative stress, and apoptosis.

## 1. Introduction

Lung cancer is still the deadliest cancer globally, and the World Health Organization estimates that 2.09 million new cases are reported each year, with 1.76 million deaths (18.4 percent of all cancer deaths) [1]. Non-small cell lung cancer (NSCLC) accounts for over 85% of lung cancer cases, and its incidence is increasing every year, seriously threatening human health [2]. 

Tyrosine kinase inhibitors (TKIs) are the majorly front-line agents for treating NSCLC over platinum doublet chemotherapy [3]. Resistance to chemotherapy develops in many patients, and more than 20 percent of NSCLC patients show epidermal growth factor receptor (EFGR) mutations [4,5]. Erlotinib and Gefitinib, which are the first-generation EGFR targeted TKIs, bind to EGFR reversibly, leading cells to acquire resistance by inducing mutations that enhance the affinity to Adenosine triphosphate (ATP) (T790M-mutation), resulting in decreased interaction with its receptors following therapy [6]. Afatinib and Dacomitinib are second-line EGFR TKIs that bind to EGFR permanently and show beneficial effects in cancer therapy, but there have been toxicity concerns due to elevated wild-type EGFR off-target binding [7]. Osimertinib (OSM) is a third-generation EGFR-TKI, irreversibly binds EGFR protein activating mutations (such as L858R and Exon19 del) and also targets EGFR TKIs resistant mutations that reduce interaction with wild-type EGFR [8]. The EGFR C797S mutation has been linked to OSM resistance by enhancing receptor affinity to ATP. Additionally, the combination therapy with various TKIs has been suggested as an excellent way to combat resistance, though there are some drawbacks, such as enhanced toxicity [9]. Even though radiotherapy and chemotherapy can help advanced patients to improve their survival rate [10,11,12,13,14,15,16], these approaches are toxic to normal cells resulting in impaired immunity, bone marrow suppression and neurotoxicity [17]. Molecular targeted therapy has gradually become a new choice because of its low dosage, remarkable effect, strong specificity, and low side effects [18].

Several reports suggested that OSM inhibits the activation of several downstream pathways, such as RAS/RAF/MAPK and PI3K/AKT, and regulates different cellular processes, including DNA synthesis and proliferation [19]. Cell cycle and apoptosis regulator protein 1 (CARP-1/CCAR1) is a perinuclear phosphoprotein that co-activates the anaphase-promoting complex/cyclosome (APC/C), an E3-ubiquitin ligase, which affects cell cycle and tumor growth [20,21]. Through p53 co-activation, it also regulates chemotherapy-induced apoptosis [21]. CARP-1 functional mimetics (CFMs) inhibit cell growth in various cancer cells and cause apoptosis via lowering CARP-1 binding to the APC/C component APC2 [22]. Previous research on CARP-1 functional mimetics has primarily investigated their role in CARP-1 signaling, ignoring their ability to suppress EGFR activation. According to molecular docking studies, both CFM4.16 and CFM4.17 have been shown to bind with EGFR’s ATP binding site [6]. This is consistent with the work of other investigators who have demonstrated that compounds with the ability to target numerous components in the EGFR signaling cascade are more inhibited than those with only one target [23].

Many anti-cancer drugs are ineffective due to high interstitial pressure or tumor stromal barriers. In addition to its role in decreasing tumor interstitial barriers, telmisartan (TLM) has also been shown to aid in the delivery of nanoparticles and liposomes to tumor cells [24,25,26,27,28,29,30,31]. TLM promotes the peroxisome proliferator-activated receptor (PPAR) pathways by inhibiting PI3K signaling [32,33]. In our laboratory, we have demonstrated that TLM could enhance the anti-cancer effects of sorafenib and CFM 4.16 in the rociletinib-resistant H1975 NSCLC xenograft model, lowering the protein expression of p-EGFR/EGFR, Nanog, Sox2, Oct4, pMET/MET, TGF-beta, and MMP9 while raising the expression of E-cadherin protein [34].

Gene mutations are a substantial barrier to lung cancer treatment, and the ability to directly measure the expression levels of molecular drug targets and profile the activation of key molecular pathways allows the personalized prioritization of all molecular-targeted therapies [35]. For high-throughput quantitative transcriptomics, it has been observed that RNA sequencing is the most reliable tool [36]. Our studies used RNA seq analysis to investigate the downstream targets contributing to cancer cell growth in NSCLC.

A thorough understanding of molecular communication will provide new insights into the molecular process behind the disease’s medication action. Proteomics, a powerful method for a detailed analysis of protein changes in response to medication therapy, has been widely used to investigate molecular pathways and identify anti-cancer therapeutic targets [37]. A recent study using proteomic analysis observed potential tyrosine kinase inhibitor (OSM) sensitivity indicators in EGFR-mutated lung cancer and identified novel targets for future therapy options [38]. In our studies, proteomics was used to explore the expression of all the proteins in H1975 tumor xenografts treated with various drugs and formulations.

EGFR TKIs are still the leading therapy for a substantial percentage of NSCLCs, and the need for resistance to TKIs remains a critical breakthrough. Herein, we hypothesize that OSM (i.e., which targets EGFR T790M mutation and inhibits activation of AMPK/Lamin-B2/MAPK and PI3K/AKT) in combination with CFM 4.17 NLPFs (i.e., CARP-1 signaling and EGFR activity is inhibited by interacting with EGFR’s ATP binding site) and TLM (i.e., disrupts tumor stromal barriers and leads to enhanced permeation of drugs) will provide superior anti-cancer effects in NSCLC, and by using RNA sequence and the quantitative proteomics, we can identify novel targets that have a role to play in tumor regression.

## 2. Materials and Methods

### 2.1. Materials

CFM 4.17 was synthesized by Otava chemicals (Concord, ON, Canada), DMSO, Tween 80, Ethanol, PBS was obtained from VWR International, LLC, (Radnor, PA, USA). All additional components and reagents were bought from Sigma-Aldrich (St. Louis, MI, USA). Cell Signaling Technology provided all primary and secondary antibodies utilized in our research.

### 2.2. Formulation of CFM 4.17 Lipid Formulation (CFM-F)

CFM-F was formulated in our laboratory, thereby using an already published procedure that consists of a melt dispersion process (optimized with Design Expert and MATLAB utilizing the Box–Behnken developed surface response methodology). CFM-F showed enhanced efficacy and increased oral bioavailability [6].

### 2.3. Cell Culture

The NSCLC cell lines H1975 (E746-A750 deletion) and HCC827 were purchased from ATCC. The cells were cultured in RPMI-1640 media with 10% heat-inactivated fetal bovine serum (FBS) and (5000 units/mL penicillin, 5 mg/mL streptomycin, and 10 mg/mL neomycin; GIBCO) at 37 °C in 5% CO_2_. 

### 2.4. H1975 Xenograft Model of Non-Small Cell Lung Cancer

The Institutional Animal Use and Care Committee of Florida Agricultural and Mechanical University evaluated and approved all animal experiment procedures as per the NIH standards and all applicable national legislation. Mice were randomly divided into 5 groups, 5 in each group. The H1975 xenograft NSCLC model was developed by injecting 2.5 million H1975 cells (in a 1:1 ratio, suspended in matrigel) into the right flank of athymic female nude mice (Foxn1nu; 20–25 grams’ body weight, 5–6 weeks old). When the tumor volume reached 1500 mm^3^, the animals were treated for 2 weeks with CFM-F (40 mg/kg body weight) and OSM (25 mg/kg body weight) alone. For the CFM 4.17 solution, TLM, and OSM combination, animals were pre-treated with TLM (10 mg/kg body weight) three times per week, followed by two weeks of CFM4.17-solution (40 mg/kg body weight) and OSM (25 mg/kg body weight). For the TLM, CFM-F, and OSM combination, animals were given TLM (10 mg/kg body weight) three times a week for two weeks before receiving CFM-F (40 mg/kg body weight) and OSM (25 mg/kg body weight) for two weeks. During the course of the drug treatments, tumor volumes were measured twice a week. The digital vernier calliper was used to measure the width and length of the tumor. The formula used to calculate the tumor volume (TV) was: TV = ½ ab^2^, where ‘a’ and ‘b’ denotes the tumor’s length and width, respectively. The animals were then monitored regularly for their health and mobility, and when the tumor burden increased beyond 6000 mm^3^, the animals were sacrificed, and tumor and blood sample was collected from all animals for proteomic, RNA-seq, and Western blot experiments. Throughout the treatment, the tumor volume was measured twice weekly. The blood samples were further processed to collect the serum, then processed for TotalSeq analysis.

### 2.5. RNA Sequencing and Data Analysis 

The manufacturer’s instructions were followed to isolate total RNAs from tumor samples using the Trizol reagent (ThermoFisher, Waltham, MA, USA; 15596018). The DNase I treatment aids in the removal of traces of genomic DNA contamination in the samples. The mRNA library was created using the NEBNext Ultra RNA Library Prep Kit (NEB, E7530) and the NEBNext Poly (A) mRNA Magnetic Isolation Module (NEB, E7490). For quality control, the library was processed on an Agilent Bioanalyzer with HS DNA chip (5067-4626), and the quantification was conducted with the KAPA Library Quantification Kit (KR0405). The library was then pooled at the requisite equal molar concentrations and transferred to the Florida State University (FSU), College of Medicine Translational Laboratory for Illumina NovaSeq 6000 sequencing.

Network Analyst 3.0 software (Guangyan Zhou, Quebec, QC, Canada)was used to analyze the RNA sequencing data; genes with a count of 10%, a variance of 10%, and unannotated were separated and standardized using Log2 counts per million [39]. DEseq2 was used to find the differentially expressed genes [40]. The heatmap aids in the visualization of differentially expressed genes and gene enriched pathways, which may be seen using the same web application. The volcano graphing was conducted using a DESeq2 data set and the log10 (FDR corrected *p*-value) to the log2 (fold change).

### 2.6. Proteomics

As per the manufacturer’s protocol, in-solution digestion was carried out on an S-trap microcolumn (Prod # CO2-micro-80, Protifi). Briefly, 100 μg of lyophilized protein was resuspended in sodium dodecyl sulphate (5%), TEAB (50 mM) pH 8.5 and reduced by adding 1 µL of TCEP (5 mM final concentration) and incubating for 15 min at 55 °C. This was followed by alkylating the mixture by adding 1 µL of alkylating agent (Iodoacetamide, final concentration 20 mM) and incubating at RT in the dark for over 10 min. The mixture was acidified by adding 2.5 µL of phosphoric acid (final concentration ~2.5%) and vortexed thoroughly. Wash/Binding buffer (TEAB-100 mM (final) in 90% methanol, 165 µL) was added to the sample and mixed well. This mixture was transferred onto the S-Trap and placed in a 1.7 mL Eppendorf tube for flow-through (waste). Proteins were trapped onto the column by centrifuging at 4000× *g* for 30 s. Trapped protein was washed thrice with 150 µL of wash buffer (TEAB-100 mM (final) in 90% methanol). To fully remove wash/binding buffer, S-Trap columns were spun at 4000 g for 1 min and transferred to a new 1.7 mL Eppendorf tube for digestion. The protein was digested by adding 5 µg of trypsin in 20 µL of digestion buffer (100 mM TEAB) and incubating the tube at 37 °C overnight. Peptides were eluted sequentially by adding 40 µL of 50 mM TEAB in water, followed by formic acid (0.2%) in purified water and finally 50% acetonitrile in purified water and spinning the column at 4000 g for 1 min. Peptides from elution solution dried in a speed vac and dissolved at 1 µg/µL in formic acid (0.1%) and transferred into auto sampler glass vials.

The peptides were analyzed on an Exploris 480 Orbitrap mass spectrometer (Thermo Fischer Scientific, Bremen, Germany) connected to an Easy-nLC-1200 nanoflow liquid chromatography system (Thermo Scientific). One microgram of the peptide was loaded onto a 2 cm trap column (nanoViper, 3 μm C18 Aq) (Thermo Fisher Scientific). The samples were then analyzed on the 100 C18 HPLC Analytical Column (Acclaim™ PepMap™, 0.075 mm internal diameter, 2 mM C18 particle size, and 150 mm long Cat# 164534) using a 180 min linear gradient of buffer B (90% acetonitrile and 0.1% formic acid) at the flow rate of 300 nL/min. Full MS scans were obtained in the range of 350–1700 *m*/*z* at a resolution of 60,000 with a threshold intensity of 5000 and dynamic exclusion of 20 s using the topN method, taking the MS2 of the top 40 ions at 15,000 resolution.

Proteomic raw data were acquired from mass spectrometry by data-dependent acquisition (DDA) method and analyzed by Proteome Discoverer software (Version 2.5, (Thermo Fisher Scientific, Waltham, MA, USA)) using the Mascot software search engine to search against the uniport homo sapiens database [41,42]. The following criteria were applied to obtain differentially expressed proteins: (a) peptides with peptide score ≥ 10; (b) high protein false discovery rate (FDR) confidence < 0.01; and (c) unique peptides after digestion, and a *p*-value at ≤0.05 was used for protein grouping and significantly differentially expressed proteins were identified by setting the threshold fold change value  ≥ 1.5. Differentially expressed proteins were organized into different groups with the approach: biological process and molecular functions using Gene Ontology (GO) assignments and Kyoto Encyclopedia of Genes and Genomes (KEGG) pathways by DAVID software [43].

### 2.7. RNA Isolation

Total RNA was extracted from tissues using TRIzol reagent (Invitrogen, California, USA) and purified using an RNeasy Mini kit (Qiagen, Germantown, MD, USA). Each sample’s A260/280 absorbance ratio was measured using a Nanodrop spectrophotometer to evaluate its RNA quality and integrity (ND-1000).

### 2.8. Reverse Transcription and RT-qPCR

To examine the mRNA levels of specific genes, cDNA synthesis from total RNA was performed according to the manufacturer’s instructions using the Maxima H Minus First-strand cDNA Synthesis Kit (Thermo Fisher Scientific, Waltham, MA, USA). Various gene primers (Lamin B2, SOX2, STAT3, NFKB, SOD, MRC-1, and Histone 1 were purchased from Integrated DNA Technologies, Inc. (Redwood City, CA, USA) (Table 1). Quantitative PCR was used to detect gene expression using SsoAdvanced^TM^ Universal SYBR Green Supermix (Bio-Rad) and the CFX96 TouchTM Real-Time PCR Detection System (Bio-Rad Laboratories). Post amplification, a melt curve analysis was used to determine the reaction’s specificity. The whole mean expression level of both 18S rRNA and GAPDH genes was used as a reference for comparison when assessing relative mRNA expression using the comparative Ct (∆Ct) technique.

### 2.9. TotalSeq Assay for Serum EVs

A thawed mouse serum sample was then mixed with sterile-filtered PBS (1:1) and centrifuged at 10,000× *g* for 15 min to remove debris. For ultracentrifugation, the collected serum was further diluted with 1 mL PBS (particle-free) and centrifuged for one hour at 100,000× *g*. The serum was further diluted and combined with an 8% polyethylene glycol (PEG) solution for 30 min for the Extra PEG procedure. The pellet was resuspended in PBS after a 3000× *g* centrifugation and purified further with ultracentrifugation at 100,000× *g* for 1 h. They were all resuspended to their original volume to make the ultracentrifuged pellets comparable. The isolated EVs were used for TotalSeq assays.

Protein from the EVs sample was lysed with 0.1 percent SDS for the TotalSeq antibody assay. One microgram of EV protein was blotted onto a nitrocellulose membrane strip. On the same strip, 1 microliter of 2.5% casein blocking buffer (sheared salmon sperm ssDNA (100 g/mL and 0.05 percent tween-20 in PBS) was blotted and air-dried. Then, the strip was placed in a 1.7 mL Eppendorf tube, and a casein-blocking buffer was used for blocking for 1 h at RT. TotalSeq-A antibodies were used in this assay including TotalSeq-A0404 anti-human CD63 Antibody (353035), TotalSeq-A0132 anti-human EGFR Antibody (352923), TotalSeq-A0190 anti-mouse CD274 (PD-L1) Antibody, TotalSeq-A0373 anti-human CD81 (TAPA-1) Antibody (349521) in 100 µL casein blocking solution, a dilution of 1:2000, a TotalSeq-A antibody pool was added and incubated overnight at 4 °C. The strips were washed 5 times with PBST (0.05% tween 20 in PBS) and one time with sterile water. Using absorbent paper, excess liquid was collected from the strip and then transferred to a fresh PCR tube. The extension mix consisted of 1X buffer 2, 1 U Klenow enzyme, dNTP, and 3’-Adaptor (500 nM working concentration). Fifteen microliters extension mix was added to the fresh PCR tube to immerse the strip thoroughly. The PCR tube was then incubated for 5 min at RT before being heat-inactivated for 5 min at 95 °C on an Eppendorf PCR machine. In a 15 µL qPCR experiment, TotalSeq DNA full-length products were measured using TotalSeq forward primer and universal R primer. For all of the TotalSeq antibodies that had been tested, the supernatant was utilized as a qPCR template. In a 15 µL qPCR run, the TotalSeq DNA full-length products were measured using TotalSeq forward primer and universal-R primer [44].

### 2.10. Western Blot Analysis

The whole-cell lysates were prepared from tissues in radioimmunoassay buffer (RIPA) (Cell Signaling, Danvers, MA, USA), which consists of 1:100 protease and phosphatase inhibitors. The supernatant was recovered after centrifuging the tissue homogenates at 10,000× *g* for 20 min at 4° C. The bicinchoninic acid (BCA) assay was used to estimate protein levels. The samples (40 µg) were loaded on a precast gel with 10% SDS-PAGE (Mini-PROTEAN^®^ TGX™ Precast Gels) at 80 V, 100 mA for 2 h. The proteins were then transferred into the PVDF membrane (Bio-Rad Laboratories, Hercules, CA, USA) and further blocked with 3% BSA PBS-T for 1 h at RT. The blot was then incubated with primary antibody (Table 2, 1:1000) overnight and washed thrice with 10 mL of PBS-T for 10 min. The blot was then incubated for 1 h at room temperature with the secondary antibody (1:8000). The blots were washed three times with PBS-T for ten minutes each time and then incubated with SuperSignal West Pico Chemiluminescent substrate, and pictures were recorded with a Chemidoc. The blots were also quantified using the NIH ImageJ software’s densitometry.

### 2.11. Statistical Analysis

The mean ± standard error is used to describe all of the data presented. GraphPad Prism version 5.0 (Dr. Harvey Motulsky, San Diego, CA, USA) was used to evaluate a significant difference between the treatment groups using either a Student *t*-test or a one-way ANOVA. When the one-way ANOVA demonstrated statistical significance, Bonferroni’s multiple comparisons test was used for post hoc analysis. Statistical significance was defined as a *p*-value < 0.05.

## 3. Results 

### 3.1. Effect of TLM_CFM-F_OSM on Tumor Volume in the In Vivo Mouse Model

**Figure 1:**
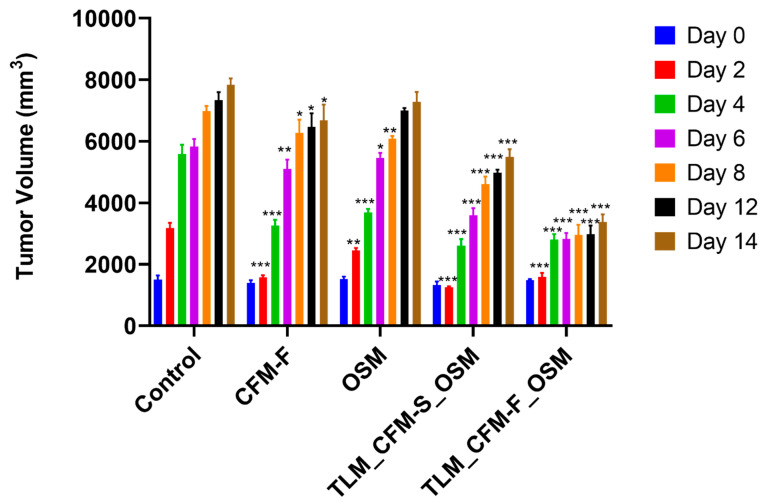
The effect of Telmisartan, CFM-F, and Osimertinib on tumor volume in experimental NSCLC. Histogram demonstrating the H1975 tumor volumes in athymic nude mice after treatment with Osimertinib (OSM), CFM4.17 lipid formulation (CFM-F), CFM4.17 solution (CFM-S), Telmisartan (TLM) and their combinations. Data were represented as the mean ± SD of three separate experiments (*n* = 3). *** *p* < 0.001, ** *p* < 0.01 and * *p* < 0.05 vs. control.

After the 14th day post-treatment, TLM_CFM-F_OSM (*p* < 0.001) and TLM_CFM-S_OSM (*p* < 0.001) combination treatment group substantially reduced the tumor volume when compared to the control, as shown in Figure 1. Further, we observed that TLM_CFM-F_OSM demonstrated a superior anti-cancer effect in reducing the tumor burden compared to TLM_CFM-S_OSM (*p* < 0.05). However, when compared to normal control, OSM and CFM-F did reduce the tumor volume (*p* < 0.05) on the 14th day (Figure 1).

### 3.2. RNA Sequencing and Differential Gene Expression Analysis in Lung Cancer

**Figure 2:**
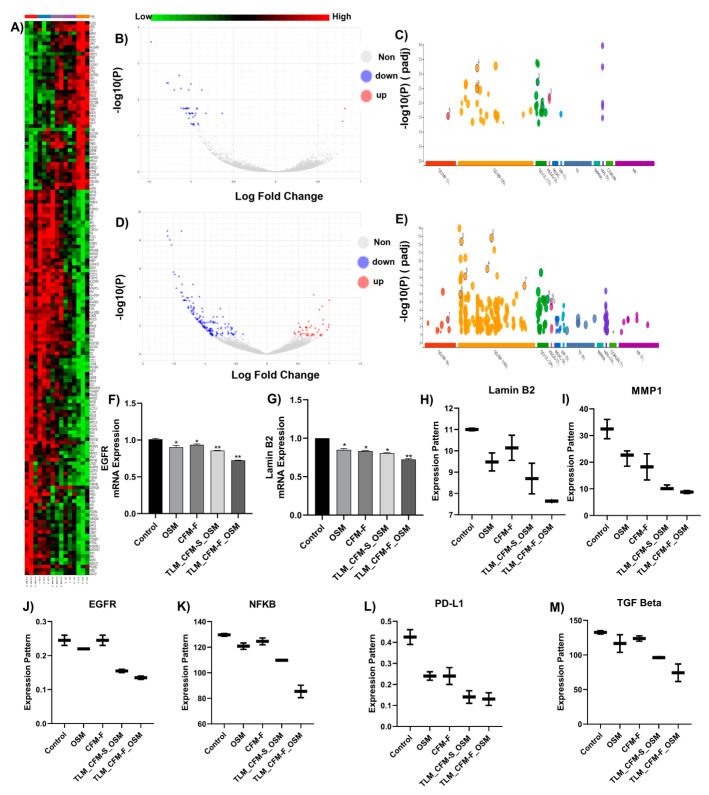
RNA-Seq identified differential mRNA expressions and their RTPCR validation in NSCLC tumor tissues isolated from athymic nude mice (**A**) Heat map illustrations of hierarchical clustering analysis of differentially expressed mRNA in tissues of control and treated H1975 Xenograft mice. Representative volcano plots of differentially expressed genes (DEGs) in between (**B**) control and TLM_CFM-F_OSM groups, (**C**) control and TLM_CFM-S_OSM groups. Representative Gene ontology (GO) and Kyoto Encyclopedia of Genes and Genomes (KEGG) annotation analysis in between (**D**) control and TLM_CFM-F_OSM groups and (**E**) control and TLM_CFM-S_OSM groups. Representative bar graphs show RT-PCR analysis of (**F**) Lamin B2 and (**G**) EGFR. Representative box plots show transcriptomic expressions of (**H**) Lamin B2, (**I**) MMP1, (**J**) EGFR, (**K**) NFKB, (**L**) PD-L1, and (**M**) TGF beta. Data were represented as the mean ± SD of three separate experiments (*n* = 3). ** *p* < 0.01, * *p* < 0.05 vs. control.

When compared to normal control tissue, RNA sequencing suggested differential regulation of numerous genes after various treatments. The determination of differentially expressed genes (*p*-value < 0.05 and FC > 1.0) between normal control and treated tissues was conducted using a heatmap (Figure 2A), which demonstrated that 950 genes were upregulated, and 1240 were downregulated after treatment. The linkage of biological pathways was determined using the KEGG pathway analysis, demonstrating differentially elevated genes. According to KEGG pathway analysis, differentially expressed genes after therapy were found to be engaged in many pathways, including spliceosome, metabolic, immunological, inflammation, mitochondrial function, apoptosis, RNA transport, and signaling. Among these, metabolic pathways (AMPK), immunological pathways (PD-L1), mitochondrial function (SOD), inflammation pathway (NFKB, STAT3, TGF beta), and apoptotic pathways (Lamin-B2, Macrophage mannose receptor 1) drew our attention because of their significance in cancer mediation shown in Figure 2. RNA seq data revealed that TLM_CFM-F_OSM induces downregulation of Lamin B2, MMP1, EGFR, NFKB, PD-L1, and TGF-beta genes. TLM_CFM-F_OSM treatment induced downregulation of Lamin B2 (i.e., 1.4-fold lower), MMP1 (i.e., 3.6-fold), EGFR (i.e., 1.8-fold), NFKB (i.e., 1.4-fold), PD-L1 (i.e., 3.46-fold), and TGF beta (i.e., 2.33-fold), in comparison to control (Figure 2H–M).

### 3.3. Validation of Differentially Expressed Transcripts via qRT-PCR

**Figure 3:**
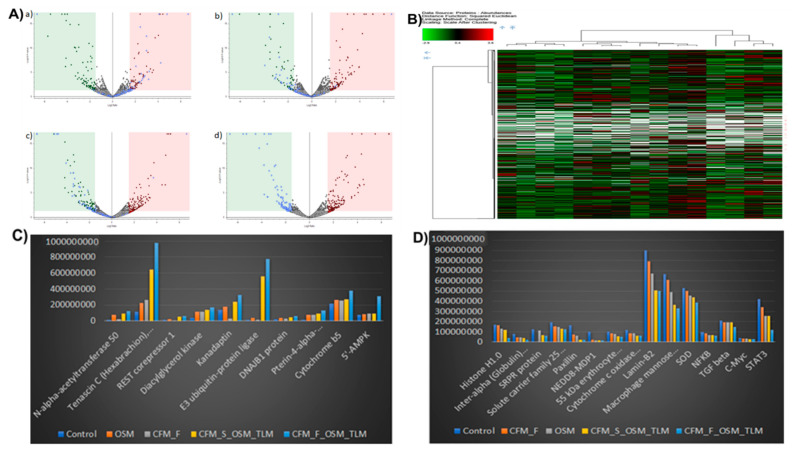
Proteomic identified differentially expressed proteins (DEPs) in lung cancer after treatment. (**A**) Representative Volcano plots of DEPs in between (a) Control and Osimertinib (OSM) groups, (b) Control and CFM4.17 nanolipid formulation (CFM-F), (c) Control and CFM-F_OSM_ telmisartan (TLM) combination, and (d) Control and CFM4.17 solution (CFM-S) OSM_TLM combination (**B**) Representing illustrations of hierarchical clustering analysis of differentially expressed proteins in control and treatment groups and proteins with the highest abundance alterations. (**C**) Schematic representation showing proteins with the largest overall increase in expression and (**D**) the proteins with the greatest reduction in expression upon treatment are represented.

We performed qRT-PCR to validate RNA-Seq data and proteomics data. Here, we selected genes that showed a highly differential expression in the treatment group compared to the control group. The qRT-PCR showed that Lamin B2 (i.e., 1.33-fold) and EGFR (1.38-fold) were significantly down-regulated (*p*  <  0.05) in the TLM_CFM-F_OSM group compared with control, not only at the proteome level (Figure 3) but also at the transcriptome level (Figure 2). As compared to the control group, every treatment group (OSM, CFM-F, and TLM_CFM-F_OSM) significantly downregulated the EGFR and Lamin B2 mRNA expression level (*p* < 0.05) but did not show significant difference across different treatment groups (Figure 2F,G). At the transcriptional level, genes showed variable expression, which would lead to changes in their protein expression.

### 3.4. Proteomics and Differential Gene Expression Analysis in Drug-Treated H1975 Tumors

Briefly, 4299 proteins were identified, and among those, 3948 proteins were quantified under both the control and treatment groups. The statistical significance level was set at *p* < 0.05 with the treatment/control group (considerably equal or greater than 1.5-fold, adjusted with the *p*-value). This parameter gave us 212 (down) and 184 (up) proteins in OSM, 175 (down) and 221 (Up) proteins in CFM-F, 214 (down) and 261 (up) proteins in TLM_CFM-F_OSM, 188 (down) and 224 (up) proteins in TLM_CFM_F_OSM when compared with the control (Figure 3A). The quantitative proteome data were used for hierarchical clustering, and the biological functions of H1975 samples are shown in Figure 3B. The functional alterations in the treatment group were determined using the Kyoto Encyclopedia of Genes and Genomes (KEGG) enrichment analysis. Differentially expressed proteins were organized into different groups using DAVID software. KEGG enrichment analysis software was used to identify differentially expressed proteins (DEPs) and those significantly enriched KEGG pathways (based on *p*-value). The upregulated and downregulated proteins in the treatment group led to various pathways: spliceosome, metabolic, inflammation, immunological, and RNA transport. Based on all the pathways given, SOD, NFKB, TGF beta, C-Myc, STAT3, Lamin-B2, Macrophage mannose receptor 1, and Histone H1.0 proteins attracted attention, which was also observed in RNA-seq, and they have also been implicated in mediating lung cancer (Table 3).

The treatment groups were analyzed individually for differentially expressed proteins with a threshold limit of 1.5-fold change. The conclusive results of the upregulated and downregulated proteins from the treatment groups and the control group are listed in Table 2. Among these, only 4–7% were identified, and differentially expressed proteins were commonly regulated in all the treatment groups and had a high abundance ratio. The high abundance of upregulated proteins is shown in Figure 3C and also the downregulated proteins in Figure 3D. The TLM_CFM-F_OSM group showed significantly downregulated proteins; Lamin B2, Macrophage mannose receptor 1, Histone H1.0, SOD2, TGF-beta, NFKB, C-Myc, STAT3, NEDD8-MDP1, Solute carrier family 25, Paxillin, and Inter-alpha inhibitor H4 as compared to TLM_CFM-S_OSM, CFM-F, OSM, and control group. Among high-abundance upregulated proteins, overexpression of AMPK, REST corepressor 1, DNAJB1 protein, and Cytochrome b5 were more significantly upregulated in the TLM_CFM-F_OSM group as compared to the TLM_CFM-S_OSM, CFM-F, OSM, and control group.

### 3.5. Combination of TLM_CFM-F_OSM Induces Anti-Cancer Effect via the LAMIN B2/STAT3/NF-κB Signaling Pathways in Lung Cancer

**Figure 4:**
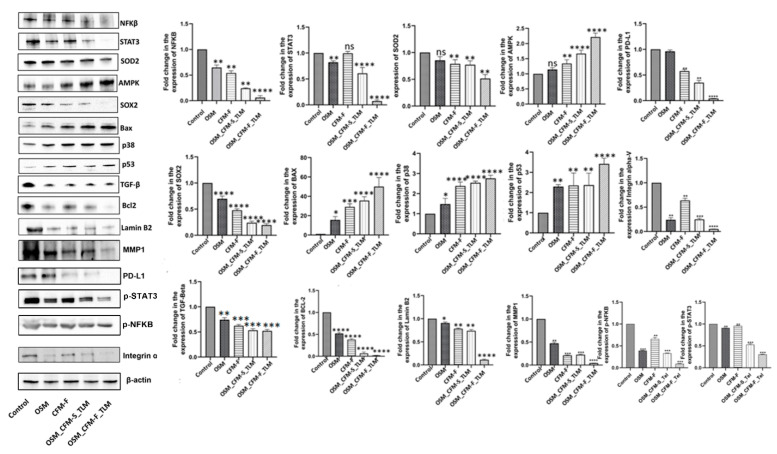
Western blot data analysis for the effect of Telmisartan, CFM-F, and Osimertinib and their combinations against H1975 lung cancer. Western blots and densitometric analysis of various proteins in the H1975 xenograft model of lung cancer. Data are representative of three different experiments and presented as mean, and error bars refer to SEM. ns *p* > 0.05 * *p* < 0.05, ** *p* < 0.01, *** *p* < 0.001, **** *p* < 0.0001 was considered significant when compared to the control.

Western blot analysis was performed to evaluate the protein expression of Lamin B2, SOD, STAT3, and NFKB. TLM_CFM-F_OSM treatment group significantly reduced the expression of Lamin B2 when compared to the control (*p* < 0.0001). TLM_CFM-F_OSM significantly downregulated the expression of Lamin B2 as compared to TLM_CFM-S_OSM (*p* < 0.01) and single treatment groups OSM (*p* < 0.05), CFM-F (*p* < 0.01). Further, the TLM_CFM-F_OSM combination significantly decreased the expression of SOD2 (*p* < 0.01), NFκB (*p* < 0.0001), STAT3 (*p* < 0.0001) protein as compared to control and single treatments (CFM-F). Collectively TLM_CFM-F_OSM demonstrated a superior anti-cancer effect, thereby decreasing the protein expression of lamin B2, SOD, STAT3, and NFKB (Figure 4).

### 3.6. Combination of TLM_CFM-F_OSM Reduced the Protein Expression of Lung Cancer Stem Cells, Fibrosis, and Migration

We examined the effects of CFM-F, TLM, and OSM on lung cancer stem cell (SOX2), migration (MMP1), and fibrosis (TGF-β) markers, since cancer stem cells, fibrosis and migration play a certain role in drug resistance development and cancer cell proliferation. It was observed that CFM-F, OSM, and TLM alone groups did not reduce the expression of TGF-β, MMP1, Oct 4, and Sox2 in H1975 lung tumors. The combination of TLM_CFM-F_OSM significantly reduced the protein expression of TGF-β (*p* < 0.0001), Sox2 (*p* < 0.0001), and MMP1 (*p* < 0.01) levels in H1975 xenografts as compared to control and TLM_CFM-S_OSM. Pre-treatment with CFM-F, followed by therapy with OSM, resulted in a significant reduction in the expression of lung cancer stem cell markers, migration, and fibrosis. Pre-treatment with TLM and CFM-F enhanced OSM sensitivity (Figure 4).

### 3.7. Combination of TLM_CFM-F_OSM Effects on the Protein Expression of Tumor Suppressor Proteins and Apoptotic Proteins

The tumor suppressor proteins (p38 and p53), AMPK, and the Bax protein all appear to affect cell apoptosis susceptibility. The combination of TLM CFM-F OSM significantly enhanced protein expression of p38 (*p* < 0.0001), p53 (*p* < 0.0001), AMPK (*p* < 0.0001) and Bax (*p* < 0.0001) in the H1975 tumors as compared to control. Here, the combination of TLM_CFM-F_OSM leads to the induction of apoptosis by increasing the protein expression of p38, p53, AMPK, and Bax in H1975 lung tumors (Figure 4).

### 3.8. The Effects of TLM_CFM-F_OSM Combination on Exosomal Markers Expression in H1975 Lung Cancer

**Figure 5:**
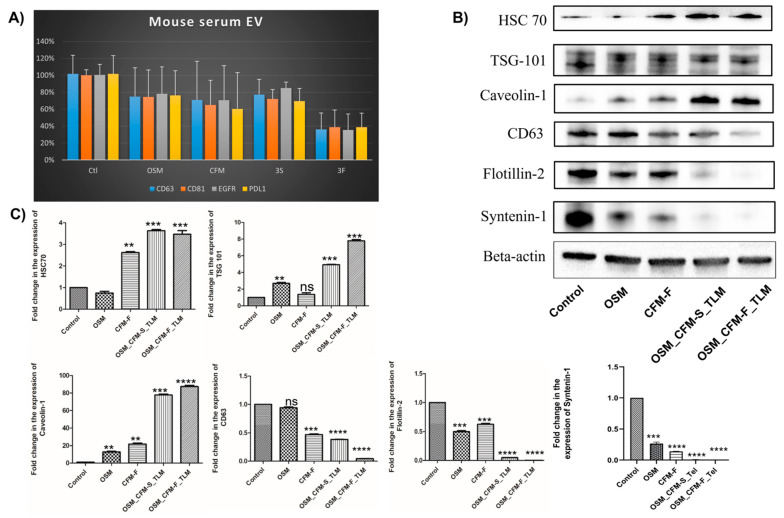
Analysis of exosomal markers in H1975 xenografts and serum samples: (**A**) Bar graph represents the TotalSeq assay in serum samples of H1975 tumor bearing athymic nude mice after treatment with different drugs and their combinations; (**B**) Representative Western blots showing expressions of exosomal protein markers after treatment in H1975 tumor tissue homogenates; (**C**) bar graphs represent the densitometric analysis of respective Western blots. Data represented as three different experiments and presented as mean ± SEM. ns *p* > 0.05 ** *p* < 0.01, and *** *p* < 0.001, **** *p* < 0.0001 was considered significant when compared to control.

Exosomes (EVs) are intercellular messengers that play a key role in cancer formation. CD63, TSG 101, Flotilin-2, calnexin, Syntenin-1, and Caveolin-1 are commonly used exosomal markers. TotalSeq assay from mouse serum EVs revealed that TLM_CFM-F_OSM induced the downregulation of exosomal marker CD63, CD81, and oncogenic proteins (EGFR and PD-L1) in lung cancer, and we further validated these results by checking the protein expression through Western blotting in lung cancer tissues after their respective treatments. Exosomal markers CD63 (*p* < 0.0001), TSG 101 (*p* < 0.001), Flotillin-2 (*p* < 0.0001), Calnexin (*p* < 0.0001), Syntenin-1 (*p* < 0.0001) were significantly downregulated and Caveolin-1 (*p* < 0.0001), HSC 70 (*p* < 0.001) protein expression were significantly upregulated as compared to control group as shown in Figure 5.

## 4. Discussion

Lung cancer with EGFR gene mutations has been observed in over 15% of NSCLC adenocarcinomas, with a frequency of around 62 percent in Asian populations [4,5,53]. TKIs are the first-line treatment for NSCLC, though resistance can develop due to hyperactivation or mutations in a variety of oncogenic proteins (in various cancers), including EGFR, fibroblast growth factor receptor, BRAF, MET, Anaplastic lymphoma kinase, vascular endothelial growth factor receptor (VEGFR), and tyrosine-protein kinase Src [54,55,56]. Different EGFR-dependent and EGFR-independent mechanisms are responsible for developing the third-generation OSM (TKI) resistance in EGFR mutant NSCLC, and the molecular mechanisms underlying TKI resistance are still being investigated [57,58,59]. There is a need to combat OSM resistance in NSCLC, and presently, there are very limited options. Hence, finding new therapeutic targets and treatment options that enhance TKI anti-cancer effects while also overcoming TKI resistance is a critical clinical need for NSCLC. To our knowledge, this is the first study to demonstrate that a combination of TLM, CFM 4.17F, and OSM has anti-cancer potential in H1975 xenografts of athymic nude mice and to understand their mechanisms using proteomics and RNA-seq studies.

The formulation of CFM 4.17 in a lipid formulation has already been established in our laboratory, and we have already demonstrated that a combination of OSM and CFM4.17 formulation inhibited the growth of H1975 cells in vitro more effectively than OSM alone, with an IC_50_ value that was 2-fold lower [6]. We did not conduct any in vitro studies for these studies since we have already published extensively with them in an earlier communication [6].

Our in vivo studies revealed that the TLM_CFM-F_OSM formulation outperformed CFM-F and CFM-S, as well as their combinations with TLM, in terms of reducing tumor burden. Based on our earlier reports, these observations are not surprising but were expected [6]. For example, in our laboratory, we demonstrated that TLM, when used in combination with CFM 4.16 and Sorafenib, significantly enhanced the anti-cancer effects of sorafenib in rociletinib resistant NSCLC xenografts (*p* < 0.01), which was attributed to disrupting tumor-stromal barriers, allowing sorafenib to penetrate deeper into tumors when administered in vivo [35]. However, in the present study, we used RNA-seq and proteomic analysis to uncover molecular changes in genes and proteins in H1975 cells to investigate the possible anti-cancer mode of action of the triple combination (TLM_CFM-F_OSM). RNA-seq (KEGG Pathway) revealed differential regulation of several genes, including metabolic (AMPK), immunological (PD-L1), mitochondrial function (SOD), inflammatory pathways (NFKB, STAT3, TGF beta), and apoptotic pathways (Lamin-B2, Macrophage mannose receptor 1). In the treatment of lung cancers, these pathways are linked to adhesion, invasion, evasion, proliferation, migration, differentiation, angiogenesis, apoptosis, and resistance to growth suppressors. In addition, we performed proteomics in the same samples to confirm the RNA-seq results and identified the upregulated (AMPK, P53, P38 and BAX) and downregulated (Lamin B2, STAT3, BCL2, SOX2, MMP-1, PDL-1, SOD, NFKB, TGF beta, and C-Myc) proteins in a sequence that matched the gene expression pattern shown in RNA sequencing data.

Adenosine monophosphate kinase (AMPK) is a bioenergetic sensor that activates in response to an elevation in the AMP/ATP ratio and is phosphorylated at Thr172 of the catalytic subunit by upstream kinases, such as liver kinase B1 (LKB1) or calmodulin kinase 1 (CAMK1), regulating metabolic homeostasis in the cell [60,61,62,63]. AMPK activation has been shown to induce tumor regression in neuroblastoma, B-cell chronic lymphocytic leukemia and breast and prostate cancer cells. This is attributed to the multiple signaling pathways, including mTOR inhibition to block protein synthesis, stabilizing p53, and cyclin-dependent kinase inhibitors to induce cell cycle arrest [64]. Similarly, we observed increased AMPK gene and protein expression levels in H1975 lung cancers in vivo after TLM, OSM, and CFM-F, and their combination showed the most superior efficacy in upregulating AMPK expression. OSM stimulates apoptosis, activating the AMPK pathway in colorectal cancer cells [65]. TLM has been demonstrated to inhibit cell growth by inducing apoptosis in a variety of cancer cell lines, including hepatocellular carcinoma (HLF cells) and gastric cancer (MKN45 cells) [66,67]. Our earlier studies have demonstrated that CFM-1, -4, and -5, CARP-1 functional mimetics, suppressed malignant pleural mesothelioma cell growth by triggering apoptosis in vitro. Further, CFM4.16 has been demonstrated to cause apoptosis via activating pro-apoptotic stress-activated protein kinases (SAPKs) such as p38 and JNK and enhanced CARP-1 production loss of the oncogene c-myc, PARP1 cleavage, and mitotic cyclin B1 [68,69]. However, while CFM 4.17-F treatment increased AMPK expression, the specific mechanism behind its activation of AMPK has to be further investigated through knockdown or knock-in studies. Based on these findings, we hypothesize that TLM_CFM-F_OSM induces apoptosis in H1975 tumors by activating AMPK.

MAPK pathways have been shown to regulate cancer growth and progression by modulating gene expression, mitosis, proliferation, metabolism, and apoptosis [70]. AMPK activity has recently been linked to p38 MAPK in several studies. AICAR, an AMPK activator that activates the p38 MAPK pathway, increases glucose uptake in skeletal muscle, but the p38 MAPK inhibitor did not affect AICAR activation [71]. Furthermore, AMPK phosphorylation of P38 MAPK induces P53 protein in various cancer cells [72]. Many cancer studies have shown that the AMPK/P38/P53 pathway increases apoptosis by regulating the expressions of BAX and caspases [73,74]. In the current study, H1975 xenografts treated with the TLM_CFM-F_OSM combination had higher levels of AMPK, p38, p53, and Bax proteins (Figure 4). Further, TLM_CFM-F_OSM significantly reduced cell proliferation and induced apoptotic cell death in H1975 lung tumors via the AMPK/p38 pathway in a p53-dependent manner compared to other treatments.

The intermediate filaments, known as Lamins, line the inner nuclear membrane, provide structural support for the nucleus and regulate gene expression [75]. Lamin B2 promotes the malignant phenotype of non-small cell lung cancer cells by interacting with micro chromosome maintenance protein 7 and Cyclin D1, both of which increase tumor motility and tumor cell epithelial-mesenchymal transition [76]. In a Drosophila laminopathy model, Chandran et al. demonstrated activating AMPK suppresses Lamin mutations and thus regulates laminopathies [77]. However, the precise relationship found between Lamin B2 and AMPK is unknown in cancer studies, and only very few studies have been conducted to investigate the role of Lamin B2 in cancer progression. The most interesting finding in our study is that Lamin B2 is the most differentially expressed protein and is highly under-expressed in the TLM_CFM-F_OSM combination treatment. Based on these findings, we hypothesize that AMPK activation by TLM_CFM-F_OSM combination controls Lamin B2 expression and thus cell proliferation, migration, and invasion in H1975 tumors.

The pleiotropic transcription factor nuclear factor-κB (NF-κB) influences lung carcinogenesis, thereby upregulating genes involved in cell proliferation, metastasis, cell migration, invasion, and apoptosis suppression [78]. Although tissue heterogeneity exists in lung cancers, the samples collected from the patients always showed an increased level of NF-κB in NSCLC [79]. In line with these findings, we discovered increased NF-κB gene and protein expression levels in H1975 tumors in the current study. Interestingly, the combination of TLM, CFM 4.17, and OSM outperformed their individual treatments in decreasing NF-κB expression in H1975 tumors. In a recent study, Jiang et al. revealed that NCI-H1975/OSM-resistant cells were highly dependent on the NF-κB pathway for survival; the treatment with the NF-κB pathway inhibitor BAY 11-7082 or genetic silencing of p65 resulted in a significantly greater number of cell deaths when compared to parental NCI-H1975/OSM resistant cells [80]. The same study demonstrated that OSM resistance was achieved through TGFβ2-mediated epithelial-mesenchymal transition and NF-κB pathway activation. In our laboratory, we have already demonstrated that CFM-4.16 formulation in combination with sorafenib inhibited the growth of tumor xenografts formed from rociletinib-resistant H1975 NSCLC cells by inhibiting the NF-κB pathway [35] and TLM treatment significantly reduced the inflammatory and hyperproliferative changes in lung tissue after ovalbumin challenge in rats [81]. Based on these findings, we hypothesize that TLM and the CFM 4.17F increase OSM antitumor effects in H1975 cancers by decreasing NF-κB activity.

TGF-β regulates the proliferation, differentiation, apoptosis, migration, adhesion, immune surveillance, and survival of many cancer cells. According to Mingze ma et al., TGF-β promotes epithelial-mesenchymal transition in A549 human lung cancer cells via the NF-κB/nox4/ROS signaling pathway [82]. In line with these findings, the current study demonstrated considerable TGF-β expression in H1975 tumors and treatment with the TLM_CFM-F_OSM combination significantly reduced its expression in H1975 tumors, as evidenced by Western blotting, RNA sequencing, and proteomics analysis. TLM reduced TGF-β levels in NSCLC lung tumors, which was linked to PPAR-γ activation, VEGF, and MMP-9 inhibition, resulting in more nanoparticle penetration into the tumor [28,31,32]. As a result, TLM, in combination with any other anti-cancer drug, would be more effective in treating metastatic lung cancers. In addition, MMP-9 is involved in lung cancer invasion, metastasis, angiogenesis, and progression [83]. It negatively affects cancer immune modulation via TGF-β activation and intercellular adhesion molecule-1 shedding (ICAM-1) [84]. Indeed, we believe that the TLM_CFM-F_OSM combination’s superior anti-cancer properties against NSCLC lung tumors are due to increased TGF-β and MMP-9 expression as well as increased CFM-F penetration into the tumors in this study.

**Figure 6:**
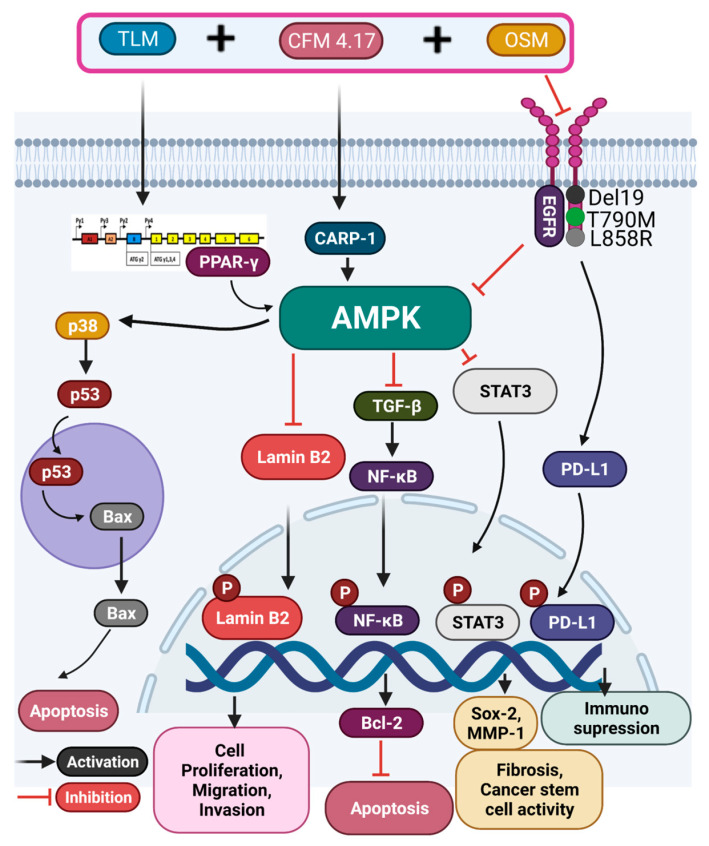
Plausible mechanism of action of CFM4.17, Telmisartan and Osimertinib combination against non-small cell lung cancers in athymic nude mice. Telmisartan activates the PPAR-γ nuclear receptor, CFM4.17 acts on CARP-1, and Osimertinib inhibits the EGFR mutated gene, increasing AMPK activity and thus regulating the p38 MAPkinase pathway, Lamin B2 protein, JAK-STAT pathway, PDL-1, and NF-κB pathway to maintain apoptosis, cancer metastasis, and immune suppression. Bcl-2: B-cell lymphoma 2, Bax: BCL2-associated X protein, CARP-1: Cell cycle and apoptosis regulatory protein 1, EGFR: epidermal growth factor receptor, CFM-F: lipid formulation of CFM4.17, MMP-1: Matrix metalloproteinase-1, OSM: Osimertinib, PD-L1: Programmed cell death 1 ligand 1, STAT3: Signal transducer and activator of transcription 3, SOX2: SRY-Box Transcription Factor 2, TLM: Telmisartan.

STAT3 is one of the potential therapeutic targets for NSCLC. The level of constitutive STAT3 activation has been linked to lung cancer metastasis, angiogenesis, and resistance to a variety of anti-cancer drugs [85]. The chemotherapeutic sensitivity of OSM against non-small cell lung cancer cells was increased when STAT3 was suppressed by chemically modified siRNAs. STAT3 and NF-κB activation and interaction are crucial in controlling cancer cell-inflammatory cell communication. NF-κB and STAT3 are critical regulators of tumor angiogenesis and invasiveness in pre-neoplastic and malignant lung cancer cells [86]. We noted a significant reduction in STAT3 expression in H1975 tumors post-treatment with the TLM_CFM-F_OSM combination, which is consistent with these reports. Based on these findings, we hypothesize that NF-κB inhibition by the TLM_CFM-F_OSM combination regulates STAT3 expression and thus tumor growth and metastasis in H1975 tumors. Collectively TLM_CFM-F_OSM affects multiple pathways, including the AMPK, NF-κB, Lamin B2, and JAK-STAT pathways, as shown in Figure 6.

EVs from cancer cells contain microRNA, long non-coding RNA, small interfering RNA, DNA, protein, and lipids, which are all being studied for use in cancer diagnosis and treatment [87]. Shimada Y et al. investigated serum exosomal PD-L1 as a quantitative marker for predicting anti-PD-1 response and evaluating clinical outcomes in NSCLC patients [88]. In line with this study, EV markers from NSCLC tumors and serum showed significantly lower levels of exosomal markers (CD63, CD81, EGFR, and PD-L1) after treatment with the TLM_CFM-F_OSM combination in the present study. According to a growing body of evidence, EVs derived from NSCLC tumors also increased PD-L1 expression and, thus, tumor development, decreased CD8+ T-cell function, and induced CD8+ T cell death [89]. Furthermore, exosomal wild-type EGFR has been shown to cause OSM resistance in NSCLC (H1975) cancers [90], and exosomal EGFR was downregulated in this study by TLM_CFM-F_OSM treatment, suggesting that combination treatment affecting exosomal PD-L1 and EGFR expression could be helpful in reversing NSCLC tumor growth and OSM resistance.

## 5. Conclusions

TLM_CFM-F_OSM showed a significant anti-cancer effect against H1975 tumor xenografts in athymic nude mice. Further, our in vivo studies with H1975 lung cancer cells demonstrated that this combination is effective through multiple pathways, including AMPK, NF-κB, Lamin B2, and JAK-STAT, which regulates mitochondrial homeostasis, inflammation, oxidative stress, and apoptosis. One novel mechanism of this triple combination in reducing the tumor burden of H1975 xenografts was the effect on serum exosome production and PDL1 and EGFR expressions. In addition, extensive molecular research is required to identify the specific molecular targets of these anti-cancer drugs for lung cancer treatment.

## Figures and Tables

**Table 1:** Primer list.

Gene	Primer
Lamin B2	F: CGGAGAGTCCTGGATGAGAC
	R: TCTTCTTGGCGCTCTTGTTG
EGFR	F: AACACCCTGGTCTGGAAGTACG
	R: TCGTTGGACAGCCTTCAAGACC
GAPDH	GTCTCCTCTGACTTCAACAGCG
	ACCACCCTGTTGCTGTAGCCAA

**Table 2:** Antibody list.

S.No	Antibody Name	Company	Catalog No.
1	Lamin B2	Cell Signaling Technology	12255
2	SOD	Cell Signaling Technology	13141
3	SOX2	Cell Signaling Technology	3579
4	EGFR	Cell Signaling Technology	54359
5	AMPK	Cell Signaling Technology	2532
6	TGF-beta	Cell Signaling Technology	3709
7	Bcl2	Cell Signaling Technology	4223
8	Bax	Cell Signaling Technology	5023
9	p38	Cell Signaling Technology	8690
10	P53	Cell Signaling Technology	2527
11	HSC 70	Santa Cruz Biotechnology	sc-7298
12	TSG 101	Cell Signaling Technology	28405
13	CD 63	Cell Signaling Technology	28405
14	Calnexin	Cell Signaling Technology	2679
15	Flotillin-2	Cell Signaling Technology	3436
16	Caveolin-1	Cell Signaling Technology	3267

**Table 3:** Proteins with high abundance values and role in cancer.

Protein Name	Role in Cancer	Expression in Treatment Group
**Histone H1.0**	Histone H1.0 overexpression in all cancer cells promotes differentiation during tumor development [45].	Downregulated
**Lamin-B2**	By upregulating demethylation of histone 3 lysine 9, Lamin B2 increases the malignant phenotype of non-small cell lung cancer cells [46].	Downregulated
**Macrophage mannose receptor 1**	Tumor-associated macrophages (TAMs) that express the multi-ligand endocytic receptor mannose receptor (CD206/MRC1) have a role in angiogenesis, metastasi, tumor immunosuppression, and recurrence [47].	Downregulated
**SOD2**	High superoxide dismutase 2 (SOD2) expression is associated with a poor prognosis at many cancer sites, the presence of metastases, and more advanced cancer [48].	Downregulated
**NFKB**	The oncogenesis process is influenced by the pleiotropic transcription factor NFKB, which upregulates genes involved in cell proliferation, metastasis, apoptosis suppression and angiogenesis [49]	Downregulated
**TGF beta**	TGF- is the most potent inducer of epithelial-mesenchymal transition in non-small cell lung cancer cells, and it is essential for the establishment of a tumor-promoting microenvironment in lung cancer tissue [50].	Downregulated
**STAT3**	Many malignancies have constitutively active STAT3, which plays a key role in tumor development and metastasis [51].	Downregulated
**AMPK**	In cancer, AMPK plays a tumor suppressor role. Activation of AMPK reduces tumor growth by targeting several tumorigenesis-related signaling pathways at various phases of tumor formation [52].	Upregulated

## Data Availability

The data that support the findings of this study are available on request from the corresponding author.
